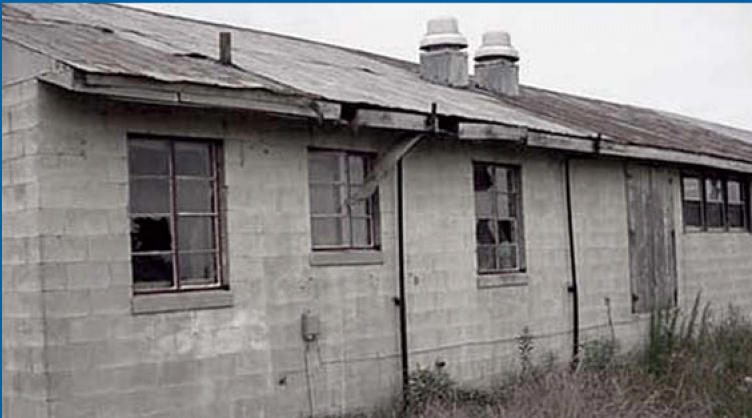# Headliners: Public Health: Inadequate Housing May Put Immigrant Farmworkers at Risk

**Published:** 2006-08

**Authors:** Tanya Tillett

Early J, Davis SW, Quandt SA, Rao P, Snively BM, Arcury TA. 2006. Housing characteristics of farmworker families in North Carolina. J Immigr Minor Health 8(2):173–184.

Even though rates of substandard housing for the general U.S. population are relatively low, percentages for subpopulations such as immigrants are disproportionately high. In this report NIEHS grantee Thomas A. Arcury and colleagues at Wake Forest University School of Medicine describe specific housing conditions for immigrant farmworker families in North Carolina, and identify housing features that leave the occupants vulnerable to environmental exposures.

Inadequate housing is a known contributor to poor health. Overcrowding and lack of proper sanitary facilities can lead to higher incidences of infectious disease, and substandard housing with structural or electrical problems poses the danger of physical injuries and exposure to toxic substances such as lead and polychlorinated biphenyls. Inadequate housing can also have negative effects on psychological health.

The researchers analyzed data from four surveys of North Carolina farm-worker communities conducted in 2001 and 2003 by specially trained interviewers fluent in Spanish. From the survey responses, the researchers documented housing conditions for 234 households of immigrant Latino farmworkers, most of whom (90%) had immigrated from Mexico. All participating houses had at least one adult farmworker and one child. The investigators considered three main features in the participants’ houses that could affect their health: characteristics of the dwelling itself, characteristics of the people comprising the household, and housekeeping behaviors.

Compared to 7% of the U.S. population as a whole, 54–70% of the immigrants surveyed lived in mobile homes, and many (36–46%) lived in crowded conditions. Most of the homes had only one bathroom. Most respondents did not own their own dwellings, and therefore had no control over how often necessary repairs were addressed.

Many respondents reported living in households that included more than the traditional nuclear family (two adult parents and children). Most reported that they dusted, swept, and mopped their floors daily. Many did not own a working vacuum cleaner, and cleaned carpets with water or brooms. Over a third of respondents did not have a working clothes washer or dryer in the home, and up to 44% lived adjacent to agricultural fields; both conditions potentially left them susceptible to pesticide exposure.

The authors conclude that the health of these families may be at risk due to inadequate housing. They add that research focusing on farmworker perceptions and decisions regarding their housing situations as well as more information on housing availability, affordability, and quality is needed.

## Figures and Tables

**Figure:**